# Real-Life Experience of Hepatitis C Treatment with Direct-Acting Antivirals in Genotypes 2 and 3

**DOI:** 10.5152/tjg.2025.24751

**Published:** 2025-09-10

**Authors:** Sibel Yıldız Kaya, Behice Kurtaran, Nefise Öztoprak Çuvalcı, Yusuf Önlen, Mehmet Reşat Ceylan, Esra Zerdali, Nagehan Didem Sarı, Ahsen Öncül, Nurettin Erben, Ayse Batirel, Dilara İnan, Süheyla Kömür, Figen Sarıgül Yıldırım, Hasibullah Yaqoobi, Ferit Kuscu, Rahmet Guner, Fehmi Tabak, Hep-C Türkiye Study Group

**Affiliations:** 1 Department of Infectious Disease and Clinical Microbiology, İstanbul University Cerrahpaşa Faculty of Medicine, İstanbul, Türkiye; 2 Department of Infectious Disease and Clinical Microbiology, Çukurova University Faculty of Medicine, Adana, Türkiye; 3 Department of Infectious Disease and Clinical Microbiology, Antalya Training and Research Hospital, Antalya, Türkiye; 4 Department of Infectious Disease and Clinical Microbiology, Mustafa Kemal University Faculty of Medicine, Hatay, Türkiye; 5 Department of Infectious Disease and Clinical Microbiology, Harran University Faculty of Medicine, Şanlıurfa, Türkiye; 6 Department of Infectious Disease and Clinical Microbiology, Haseki Training and Research Hospital, İstanbul, Türkiye; 7 Department of Infectious Disease and Clinical Microbiology, İstanbul Teaching and Research Hospital, İstanbul, Türkiye; 8 Department of Infectious Disease and Clinical Microbiology, İstanbul Şişli Hamidiye Etfal Training and Research Hospital, İstanbul, Türkiye; 9 Department of Infectious Disease and Clinical Microbiology, Eskişehir Osmangazi University Faculty of Medicine, Eskişehir, Türkiye; 10 Department of Infectious Disease and Clinical Microbiology, Kartal Dr Lütfi Kırdar City Hospital, İstanbul, Türkiye; 11 Department of Infectious Disease and Clinical Microbiology, Akdeniz University Faculty of Medicine, Antalya, Türkiye; 12 Department of Infectious Disease and Clinical Microbiology, Başkent University Adana Dr.Turgut Noyan Training and Research Center, Adana, Türkiye; 13 Department of Infectious Disease and Clinical Microbiology, Yıldırım Beyazıt University Faculty of Medicine, Ankara, Türkiye

**Keywords:** Chronic hepatitis C, direct-acting antivirals, genotype 2, genotype 3, hepatitis C virus

## Abstract

**Background/Aims:**

Despite the widespread use of direct-acting antivirals (DAAs), real-world data on treatment outcomes and predictors of response in hepatitis C virus (HCV) genotypes 2 and 3 remain limited, particularly in countries with heterogeneous patient populations such as Türkiye. This study evaluates the efficacy and safety of direct-acting antivirals (DAAs) in treating hepatitis C virus (HCV) genotype 2 (GT-2) and genotype 3 (GT-3) in Türkiye.

**Materials and Methods:**

This cohort is a multicenter, retrospective, and observational study. Data from 267 GT-2 or GT-3 patients treated with a DAA were analyzed. Treatment efficacy was assessed by sustained virological response at 12 weeks after the end of treatment (SVR), and baseline demographic, clinical, and laboratory parameters were evaluated to identify factors associated with treatment response..

**Results:**

An overall sustained virological response (SVR) rate of 95.9%, with no significant difference between GTs. The SVR rates were relatively lower in patients with cirrhosis. Prior pegylated interferon and ribavirin reduced SVR rates, particularly in males and patients with cirrhosis. The most common treatments were sofosbuvir-based regimens, which demonstrated comparable efficacy. No significant drug interactions were observed. The most commonly reported adverse events were fatigue and mild anemia, particularly in cirrhotic patients; however, these did not lead to treatment discontinuation.

**Conclusion:**

This study supports the efficacy and tolerability of DAA regimens for these HCV GTs, thereby reinforcing their role in HCV eradication.

**Main Points**

- Direct-acting antivirals (DAA)-based regimens have excellent efficacy and safety in patients with genotypes 2 and 3 chronic hepatitis C virus (HCV).
- Although the clinical response to DAA is excellent, there are still some patient groups that are difficult to treat.
- The factors that had a negative impact on sustained virological response were baseline high alanine aminotransferase value, presence of cirrhosis, history of hepatocellular carcinoma, and prior pegylated interferon/ribavirin treatment.
- With the success of new therapies, hepatitis C virus (HCV) screening is a key step toward HCV eradication.

## Introduction

Chronic hepatitis C (CHC) is still an important cause of liver-related morbidity and mortality worldwide. According to the 2015 data from the World Health Organization, the number of CHC patients was 71.1 million and its mortality rate was 475 000 people per year.^1^ With the use of new direct-acting antivirals (DAAs), the number of hepatitis C virus (HCV) patients and HCV-related deaths have decreased significantly. According to 2022 data, there were approximately 56.8 million CHC patients, while 257 000 people died due to HCV-related cirrhosis or hepatocellular carcinoma (HCC).^2^

The HCV genotypes and subtypes (1a, 1b, 1c, 2a, 2b, 2c, 3a, 3b, 4, 5, and 6) vary according to geographical regions. While genotype 1 (GT-1) is the most prevalent, genotypes 2 and 3 (GT-2 and GT-3) represent approximately 30% of HCV GTs.^3^^-^^5^ In Türkiye, GT-2 and GT-3 are less prevalent than GT-1. Although GT-2 and GT-3 have demonstrated relatively favorable outcomes with interferon-based regimens, response rates with DAAs are lower.^3^ However, treatment response is still more successful than with interferon-based treatments.

The DAAs have revolutionized HCV treatment, providing higher cure rates with shorter durations and fewer side effects compared to interferon-based therapies. The DAAs are categorized into 3 main classes: nonstructural protein 3 (NS3)/4A protease inhibitors (e.g., simeprevir, grazoprevir), which block viral protease activity; NS5A replication complex inhibitors (e.g., ledipasvir (LDV), velpatasvir (VEL)), which interfere with viral replication and assembly; and NS5B polymerase inhibitors, further divided into nucleoside analogs (e.g., sofosbuvir (SOF)), which inhibit viral RNA synthesis, and non-nucleoside analogs (e.g., dasabuvir (DSV)), which disrupt polymerase function.^2^^-^^7^

This study aimed to investigate the efficacy and safety of DAA regimens in GT-2 and GT-3 CHC patients followed in different centers across Türkiye.

## Materials and Methods

The cohort is a multicenter, retrospective, and observational study. Data of 2713 CHC patients, whose HCV RNA was detectable by molecular methods in the sera, treated with DAA in 37 different centers in Türkiye between April 2017 and December 2019 were enrolled into the Clinical Trials (clinicaltrials.gov, registration number: NCT03145844) database. The inclusion criteria are presence of detectable HCV RNA levels for at least 6 months, being 18 years or older and initiation of treatment with DAAs. The exclusion criteria are being younger than 18 years old, pregnancy, and patients with missing follow-up data.

Among 2713 patients recorded into the database; 352 (12%) were infected with GT-2 or GT-3. A total of 85 patients were excluded from the study due to incomplete or missing follow-up data. In the end, 267 patients with GT-2 or GT-3 were enrolled in the study (85%-31.8% and 182%-68.2%, respectively).

Baseline laboratory tests were conducted on all patients at the beginning of the treatment period. Abdominal ultrasonography was performed on all patients and abdominal computed tomography (CT) and/or magnetic resonance imaging were also performed in cirrhotic patients. Patients’ comorbidities were recorded and patients were questioned about the drug’s adverse reactions, including severe fatigue, sleep disturbances, depression, skin eruptions, and dyspnea. Any reports of adverse events other than these were also recorded.

The HCV RNA was analyzed at the beginning, the end of the treatment, and the 12th week post treatment. An undetectable HCV RNA level at the end of treatment was considered an end-of-treatment response (EoTR), and an undetectable HCV RNA level at the 12th week of post treatment was considered as sustained virological response (SVR). The primary endpoint of this study was SVR at week 12 after the end of treatment.

Patients were treated with one of the following regimens: glecaprevir (GLE) + pibrentasvir (PIB), SOF + ribavirin (RBV), lLDV + SOF ± RBV, paritaprevir/ritonavir (PTV/r) + ombitasvir (OBV) ± DSV ± RBV. The RBV was added to the treatment of patients with compensated/decompensated cirrhosis. The selection of a DAA regimen was guided by the reimbursement criteria set forth by the Turkish Ministry of Health at the time of study.

SPSS 21.0 (SPSS Inc.; Chicago, IL, USA) was used for statistical analysis of the data. Continuous variables are expressed as mean ± standard deviation (SD). The categorical values are reported as frequencies and percentages. The chi-squared test was used to compare categorical variables. The Mann–Whitney *U* test was used in cases where the continuous variables did not have a normal distribution. For the analysis of continuous variables with a normal distribution, the Student’s *t*-test was used. Correlational analysis was performed using Pearson’s correlation test (r) for normally distributed numerical parameters and Spearman’s Rho test (rho) was used to analyze skewed and categorical data. Statistical significance was defined as a *P*-value of less than .05.

Ethical committee approval was received from the Ethics Committee of İstanbul University Cerrahpaşa (Date: March 07, 2017, Approval No: 59491012-604.01.02). Verbal consent was obtained from all participants. The study was recorded on clinicaltrials.gov (https://clinicaltrials.gov/study/NCT03145844). The trial registry name was “Direct Acting Agents in Hepatitis C Patients (HEPCTURKEY)” and the registration number was NCT03145844.

## Results

Seventy-six (28.5%) of the patients were female and the mean age was 44 ± 18 years (range 19-85 years). The mean age of GT-2 patients was significantly higher than that of GT-3 patients (54 ± 19 vs. 38 ± 15, *P* = .001). While the number of males and females was similar in GT-2 cases (44 vs. 41), the male gender was predominant in GT-3 cases (147 vs. 35, *P* = .001). Patients’ characteristics and baseline laboratory values of the patients are mentioned in Table 1.

A total of 114 patients were identified as having a suspected contact in their medical history. Possible transmission routes included intravenous drug use (IVDU) in 97 persons, blood transfusion before 1990 in 6 persons, hemodialysis in 5 persons, dental procedures in 3 persons, a history of previous surgery in 2 persons, and a history of tattooing in 1 person. Eighty-three (87.4%) of the IVDU individuals belonged to GT-3 group. Comorbidities included chronic renal failure (CRF) (14 cases), heart disease (17 cases), hypertension (40 cases), hypothyroidism (15 cases), and type 2 diabetes mellitus (17 cases). Chronic systemic diseases were significantly higher in the GT-2 group than in the GT-3 group (44.7% vs. 13.7%, *P *= .001).

Liver biopsy was available in 153 (107 GT-3 and 46 GT-2; 57.3% of study population) patients; mean histological activity index (HAI) was 7.4 and fibrosis score was 2.4 according to Knodell histological activity index. The HAI was mild (1-5) in 17.6%, moderate (6-12) in 77.1%, and severe (13-18) in 5.2%. Fibrosis was mild (0-1) in 24.8%, moderate (2-3) in 66%, and advanced (4-6) in 9.1%. Cirrhosis was present in 27 cases with clinical and/or pathological concordance. Of these, 26 patients had Child-Pugh score A cirrhosis, while 1 had Child-Pugh score C cirrhosis. Cirrhosis was significantly more common in GT-2 than GT-3 (15.3% vs 7.7%, *P* = .044).

While 220 (82.4%) patients were treatment-naive, 47 (17.6%) had previously been treated with pegylated interferon and RBV (peg-IFN/RBV). The majority of them (73%) were relapsed and the remaining were nonresponders. The SVR was significantly lower in patients received peg-IFN/RBV compared with those who did not (89.4% vs. 97.3%, *P *= .28). One patient was being experienced with first-generation protease inhibitors (telepravir (TPV) + peg-IFN/RBV). This patient, did not respond to the combination of TPV + peg-IFN/RBV treatment, was treated with PTV/r + OBV ± DSV (PROD) ± RBV and SVR was achieved.

The baseline mean viral load was 4.09 x 10^6^ copies/mL. Treatment regimens included SOF + RBV (223 cases), LDV + SOF ± RBV (27 cases), GLE + PIB (11 cases), and PROD ± RBV (6 cases) (Table 2). Weight-based RBV was added to the compensated/decompensated cirrhotic patients. No significant differences were observed between the regimens and treatment responses (*P *> .5) (Table 3). The online DeLong Test (http://vassarstats.net/roc_comp.html) was also used to evaluate significance of the difference between the areas under 2 independent ROC curves, and no statistical significance was found.

The EoTR and SVR were 98.1% and 95.9%, respectively. The SVR was 95.3% in GT-2 cases and 96.2% in GT-3 ones. According to multivariate logistic regression analysis, age, cirrhosis, and previous treatment history were statistically significant factors affecting SVR. The analysis revealed that cirrhosis significantly decreased the likelihood of achieving SVR (OR = 35.307, *P* = .023), while previous treatment history borderline decreased the chance of SVR (OR = 5.418, *P *= .069). Age and ALT level had no significant impact on SVR outcomes (*P* = .985 and *P* = .145, respectively). Additionally, gender was borderline significant, with females having a 13.5-fold higher likelihood of achieving SVR (OR = 13.506, *P* = .058). No significant difference was observed between GT-2 and GT-3 in terms of SVR (*P *= .893). Patients who did not achieve SVR were predominantly male (90.9%) and had higher baseline ALT levels. Mean baseline ALT levels are 71 (±62) IU/L in SVR achievers and 98 (±185) IU/L in nonresponders. Cirrhosis (33.3%) and prior peg-IFN/RBV treatment (45.5%) were also more common in this group.

The SVR was lower in patients with cirrhosis at baseline compared to non-cirrhotic patients (89% vs 97%, *P* = .027). The SVR could not be achieved in 2 patients with a history of HCC. Although no biochemical tests were identified that have an impact on SVR, a statistically significant difference was observed between baseline ALT-AST levels and EoTR (*P *= .01 vs. *P* = .02). Additionally, baseline ALT (71 vs. 98, *P *= .001) and GGT (46 vs. 83, *P *= .011) were significantly different between patients with and without SVR. In the correlation analysis, cirrhosis (*P *= .027, rho = 0.140), history of HCC (*P *= .001, rho = 0.253), and previous treatment experience (*P *= .013, rho = 0.152) have a statistically significant correlation with SVR.

In the response of CRF patients, who are notoriously difficult to treat with IFN-based regimens, SVR was achieved in all 13 CRF patients, 5 of whom were on hemodialysis.

A single patient coinfected with HIV achieved SVR. Seven patients were coinfected with HBV (4 with chronic HBV infection and 3 with HCC), and SVR was reached in 5. One patient with EoTR, has relapsed subsequently, and the patient was re-identified as GT-3 through repeated genotyping. One patient died on treatment due to complications associated with a femur fracture.

The drugs were well-tolerated, and no severe adverse events were reported. The most frequently reported side effects were fatigue (7%), insomnia (2%), itching (2%), headache (1%), and nausea (0.7%). Among patients who developed anemia due to RBV treatment, the RBV dose was modified according to the severity of the anemia. However, in none of them, RBV was interrupted or discontinued. Adverse events were significantly more frequent in cirrhotic than non-cirrhotic patients (33% vs 13%, *P *= .001).

## Discussion

For many years, the treatment of CHC has posed a challenge for both patients and physicians, in terms of both treatment response and side effects. However, with the introduction of DAAs, the treatment of CHC offers new hope to patients and physicians. These agents, when used in combination treatment regimens, have resulted in high rates of sustained virologic response and have replaced interferon-based therapies.^2^^,^^3^^,^^6^^,^^7^

The transmission of HCV is primarily associated with direct exposure to blood through transfusions, needlestick injuries, and IVDU. People who inject drugs, men who have sex with men, and people in prison are the main groups at high risk.^8^ In the cohort, IVDU was the most prevalent route of transmission. Furthermore, both male gender and IVDU were significantly more common among the patients with GT-3. The possible reason for the predominance of this rate was the simultaneous anti-HCV screening by Alcohol and Substance Abuse Treatment Center during this clinical trials. In different cohorts, the history of IVDU was also present to be more common in GT-3 patients.^9^^-^^12^

In general, studies have shown that GT-3 is associated with a more aggressive course of the disease.^13^ In GT-3 patients, it is believed that liver steatosis and fibrosis increase with the direct effect of the virus.^13^ In long-term follow-up, GT-3 is expected to have more fatty liver disease, more advanced fibrosis, and a higher risk of HCC compared to GT-2.^14^ Cirrhosis was significantly more common in GT-2 than GT-3 (15.3% vs. 7.7%) in the cohort. It was considered that this was primarily due to the mean age of GT-2 patients was significantly higher than that of GT-3 ones (54 vs. 38 years), and therefore, the longer duration of disease. The IVDU is the leading etiology of new HCV cases, particularly in high-income countries. As IVDU is more prevalent in the young population, new HCV cases are more common in young people.^1^^,^^15^^,^^16^

Although the clinical response to DAA is excellent, there are still some patient groups that are difficult to treat. In the study, the factors that had a negative impact on SVR response were baseline high ALT value, presence of cirrhosis, history of HCC, and prior peg-IFN/RBV treatment. Although not statistically significant, 10 of the 11 patients with SVR failure were male.

A study investigating the efficacy of SOF + RBV combination in the treatment of HCV GT-2 and GT-3 infections achieved high rates of SVR, 93% and 85%, respectively. In patients with GT-3, factors such as female gender, absence of cirrhosis, young age, and low initial viral load were identified as predictors of SVR.^17^ In the study in Poland with real-life data, male sex, liver cirrhosis, BMI ≥ 25 kg/m^2^, infection with GT-3, presence of esophageal varices, concomitant diabetes, receiving previous ineffective treatment, baseline ALT activity >70 U/L, higher bilirubin concentration, and lower albumin level and platelet count were negative predictors of an SVR.^18^ However in 2019, real-life data from China indicate that high rates of SVR were achieved regardless of GT, liver status, age, gender, and prior treatment experience.^9^ Treatment of the GT-3 has more favorable outcome with IFN-based therapies than other GTs, whereas it has poor results with oral DAA treatment. Although SVR rates remain unsatisfied compared to other GTs, it is much better compared to IFN-based therapies.^3^^,^^6^^,^^17^^-^^21^ Despite previous studies, the findings indicate similar treatment responses between these GTs. The observed discrepancy may be attributed to several factors. First, a significant proportion of the cohort consisted of treatment-naive patients (82.4%), which is known to be a predictor of better response to DAAs. Second, the prevalence of cirrhosis, an established negative predictor of SVR, was lower in GT-3 patients compared to GT-2 patients (7.7% vs. 15.3%, *P *= .05), potentially mitigating its impact on SVR rates. Third, the cohort had a younger GT-3 patient population, as GT-3 is often linked to IVDU, a group that is typically younger and has a shorter disease duration. These factors may have contributed to a more favorable treatment response in GT-3 patients, minimizing the historical disparity between GTs.

As in other studies, prior HCV treatment experience was determined to be a negative factor in the study.^6^^,^^17^^,^^22^^-^^24^ The SVR was significantly lower in patients who received prior peg-IFN/RBV treatment. The DAA experience is seen as a more problematic risk factor as it may also be responsible for possible viral resistance.^17^ In this study, 1 patient had previously undergone treatment with first-generation protease inhibitors (TPV + peg-IFN/RBV) and had experienced a failure of treatment. This patient was subsequently treated with PROD, and SVR was achieved. Another statistically significant factor on SVR response was the presence of cirrhosis. The SVR was significantly lower in patients with cirrhosis at baseline compared to non-cirrhotic patients (89% vs. 97%). Despite notable advancements in treatment response in patients with cirrhosis relative to IFN-based regimens, the underlying condition of cirrhosis itself remains a significant contributing factor to the attainment of SVR.^17^^,^^24^^-^^27^

In the era of IFN-based therapies, most patients with renal insufficiency were unable to receive antiviral treatment due to side effects, and the SVR response was very low in patients who could be treated. With the introduction of oral DAA regimens, HCV treatment in patients with CRF has become much more favorable.^17^^,^^28^ In the cohort, SVR was achieved in all 15 CRF patients, 5 of whom were on hemodialysis. The SOF-based regimens were used in 13 of 15 renal failure patients in the cohort and no serious drug reactions were observed. Although there were initially hesitations about the use of SOF-based regimens in patients with end stage renal failure, they were approved for use in advanced renal failure in 2019.^28^^-^^30^

In the study, no adverse events required discontinuation or interruption of treatment. Similarly, mild adverse events that did not affect treatment persistence have been reported in previous studies.^6^^,^^19^^,^^25^^,^^29^ In patients receiving concomitant RBV, the dose of RBV had to be reduced due to RBV-related anemia. In the Polish study with real-world data, the rate of serious adverse events was significantly higher in cirrhotic patients.^17^ Likewise in this study adverse events were more frequent in cirrhotic patients. Although SOF + RBV was effective in the cohort, current EASL and AASLD guidelines recommend RBV-free regimens due to anemia risks and tolerability concerns. Pangenotypic DAAs such as SOF/VEL and GLE/PIB are now preferred for their comparable SVR rates and improved safety profile.^31^^,^^32^

Although all these DAAs may seem costly initially, they play a critical role in HCV eradication. In fact, studies have demonstrated that these therapies yield favorable long-term outcomes in terms of both cost-effectiveness and survival. A study conducted in Türkiye evaluated the economic benefits of HCV screening and treatment strategies. According to its findings, although the active screening and treatment scenario for HCV appears to incur higher costs in the first year compared to standard care, a 20-year projection demonstrated a total saving of 883 million TL. Moreover, the strategy proved effective in reducing HCV-related mortality, preventing 62.8% of expected deaths within the first 5 years.^33^ These results underscore the long-term economic and clinical advantages of implementing active screening combined with DAA therapy in HCV management programs.

In conclusion, this cohort shows that DAA-based regimens have excellent efficacy and safety in patients with HCV GT-2 and GT-3, even in the presence of cirrhosis. Global eradication of HCV is a realistic goal for the future, depending on the successful implementation of effective screening strategies to identify HCV-infected individuals.

## Figures and Tables

**Table 1.** Baseline Characteristics and Laboratory Values of the Patients

	Genotype 2 (85)	Genotype 3 (182)	*P*
Gender n (%)			**.001**
Female	41 (48.2)	35 (19.2)	
Male	44 (51.8)	147 (80.8)	
Age (years)	54 ± 19	38 ± 15	**.001**
Cirrhotic patients, n (%)	13 (15.3)	14 (7.7)	**.044**
HAI, n (%)	Total: 46 patients	Total: 107 patients	>.5
1-5	9 (19.5)	18 (17)	
6-12	34 (74)	84 (78.5)	
13-18	3 (6.5)	5 (4.5)	
Fibrosis, n (%)	Total: 46 patients	Total: 107 patients	>.5
0-1	9 (19.5)	29 (27.1)	
2-3	34 (74)	67 (62.6)	
4-6	3 (6.5)	11 (10.2)	
ALT (IU/L)	45 (IQR: 62)	55 (IQR: 57)	**.042**
AST (IU/L)	41 (IQR: 38)	47 (IQR: 33)	>.5
GGT (IU/L)	29 (IQR: 51)	39 (IQR: 35)	>.5
Albumin (g/dL)	4.31 (IQR: 0.62)	4.37 (IQR: 0.70)	>.5
Total bilirubin (mg/dL)	0.66 (IQR: 0.57)	0.70 (IQR: 0.59)	>.5
Urea (mg/dL)	25 (IQR: 18.45)	28 (IQR: 15.50)	>.5
Creatinine (mg/dL)	0.68 (IQR: 0.27)	0.74 (IQR: 0.17)	>.5
Alpha fetoprotein (IU/mL)	3.92 (IQR: 3.51)	3.6 (IQR: 3.18)	>.5
INR	1.03 (IQR: 0.14)	1 (IQR: 0.16)	>.5
HCV RNA (copies/mL)	4 724 446	3 806 392	>.5
EoTR, n (%)	84 (98.8)	178 (97.8)	>.5
Recurrence, n (%)	3 (3.5)	3 (1.6)	>.5
SVR, n (%)	81 (95.3)	175 (96.2)	>.5

Bold ones are statistically significant between 2 genotypes.

ALT, alanine aminotransferase; AST, aspartate aminotransferase; EoTR, end-of-treatment response; GGT, gamma glutamyl transferase; HAI, histological activity index; HCV RNA, hepatitis C virus ribonucleic acid; IQR, inter-quartile range; INR, international normalized ratio; N, number; SVR, sustained virological response.

**Table 2.** Number of Patients Receiving DAA Regimens by Genotype and Disease Status in the Study, Along with Guideline Treatment Recommendations for These Situations

		SOF/RBV	LDV/SOF ± RBV	PROD ± RBV	GLE/PIB	EASL 2023 (Recommended Regimens)	AASLD 2023 (Recommended Regimens)
Genotype 2	TN–NC	45	1	0	5	SOF/VEL or GLE/PIB (ribavirin‑free regimens preferred)	SOF/VEL or GLE/PIB (preferred)
	TN–C	7	3	0	0	SOF/VEL ± RBV or GLE/PIB (in compensated cirrhosis)	SOF/VEL ± RBV is recommended for cirrhotic patients
	TE–NC	16	2	2	1	SOF/VEL or GLE/PIB (if prior treatment was interferon based; if DAA failure then consider retreatment options)	SOF/VEL or GLE/PIB recommended
	TE–C	2	0	1	0	SOF/VEL/VOX is recommended for patients with prior DAA failure; if only interferon‑experienced, SOF/VEL may be used	SOF/VEL/VOX is recommended for retreatment in DAA failures
Genotype 3	TN–NC	135	13	2	5	SOF/VEL or GLE/PIB (preferred for non‑cirrhotic patients)	SOF/VEL or GLE/PIB recommended
	TN–C	4	0	0	0	SOF/VEL ± RBV (12 weeks; ribavirin may be added for cirrhotic patients)	SOF/VEL ± RBV is recommended for genotype 3 cirrhotics
	TE–NC	13	7	0	0	SOF/VEL or GLE/PIB (if previous treatment was interferon‑based; similar to TN–NC if no DAA failure)	SOF/VEL or GLE/PIB recommended
	TE–C	1	1	1	0	SOF/VEL/VOX is recommended for retreatment in DAA failures among cirrhotic patients	SOF/VEL/VOX is recommended

GLE, glecaprevir; LDV, ledipasvir; PIB, pibrentasvir; PROD, paritaprevir + ritonavir + ombitasvir ± dasabuvir; RBV, ribavirin; SOF, sofosbuvir; TE–C, treatment experienced–cirrhotic; TE–NC, treatment experienced–non-cirrhotic; TN–C, treatment naive–cirrhotic; TN–NC, treatment naive–non-cirrhotic; VEL, velpatasvir; VOX, voxilaprevir.

**Table 3.** Direct-Acting Antivirals and Sustained Virological Response Rates in Patients Infected with Genotypes 2 and 3 Chronic Hepatitis C

	Genotype 2		Genotype 3		Total Population	
	Number of Patients (n)	SVR, n (%)	Number of Patients, (n)	SVR, n (%)	Number of Patients, (n)	SVR, n (%)
SOF/RBV	70	66 (94.2)	153	148 (96.7)	223	214 (95.9)
LDV/SOF ± RBV	6	6 (100)	21	20 (95.2)	27	26 (96.3)
GLE/PIB	6	6 (100)	5	5 (100)	11	11 (100)
PROD ± RBV	3	3 (100)	3	2 (66.7)	6	5 (83.3)
Total	85	81 (95.2)	182	175 (96.1)	267	256 (95.8)

GLE, glecaprevir; LDV, ledipasvir; PIB, pibrentasvir; PROD, paritaprevir + ritonavir + ombitasvir ± dasabuvir; RBV, ribavirin; SOF, sofosbuvir.

## Data Availability

The data that support the findings of this study are available on request from the corresponding author.
